# Special Issue “Recent Advances in Thermoelectric Materials for High Efficiency Energy Conversion and Refrigeration”

**DOI:** 10.3390/ma15051672

**Published:** 2022-02-23

**Authors:** Paolo Mele

**Affiliations:** 1 College of Engineering, Innovative Global Program, Shibaura Institute of Technology, 307 Fukasaku, Minuma-ku, Saitama 337-8570, Japan; pmele@shibaura-it.ac.jp; 2 International Center for Green Electronics, Shibaura Institute of Technology, 3-7-5 Toyosu, Koto-ku, Tokyo 135-8548, Japan

It has been almost three years since I enthusiastically accepted to be guest editor for this Special Issue of *Materials*, entitled “Recent Advances in Thermoelectric Materials for High Efficiency Energy Conversion and Refrigeration”.

Thermoelectricity is a well-known phenomenon enabling the conversion of heat into electric energy without moving parts. Its exploitation has been widely considered to contribute to the increasing need for energy along with the concerns about the environmental impact of traditional fossil energy sources. In the last few years, significant improvements in the performance of thermoelectric materials have been achieved through chemical doping, solid solution formation, and nanoengineering approaches. Furthermore, the feasibility of flexible, stretchable, and conformable thermoelectric harvesters has been demonstrated and has attracted the interest of a wide audience. However, the path for practical applications of thermoelectrics still appears long.

This Special Issue of *Materials* was intended as an effort to bridge the gap between materials science and applications of thermoelectric materials. 

Originally, many topics were welcome: new thermoelectric compounds; correlation between material structure and thermoelectric properties; bulk thermoelectric ceramics, oxides, and chalcogenides; bulk thermoelectric alloys and intermetallics; organic and polymeric thermoelectrics; thermoelectric thin films, multilayers, and nanocomposites; theory and modeling; thermal transport and thermal conductivity; applications and devices based on thermoelectric materials; standardization and metrology; and more.

The five published papers reflect the original spirit of the Special Issue, going from skutterudite materials to sulfides and oxides and covering various aspects of materials preparation and characterization.

With the great hope that it will contribute to pave the way for wide diffusion of thermoelectric materials in society and daily life, I declare this Special Issue closed thanking all the colleagues and all the editorial staff of *Materials* for their great contributions and everlasting support.

